# Corneal Cross-Linking for Keratoconus and Post-LASIK Ectasia and Failure Rate: A 3 Years Follow-Up Study

**DOI:** 10.7759/cureus.19552

**Published:** 2021-11-13

**Authors:** Wassef Chanbour, Lulwa El Zein, Mohamad Ali Younes, Mohamad Issa, Pramod Warhekar, Elias Chelala, Elias Jarade

**Affiliations:** 1 Ophthalmology, Beth Israel Deaconess Medical Center (BIDMC), Boston, USA; 2 Pediatric Ophthalmology, Bascom Palmer Eye Institute, Miami, USA; 3 Internal Medicine, Faculty of Medical Sciences - Lebanese University, Hadath, LBN; 4 Ophthalmology, Hospital Foundation Adolphe De Rothschild, Paris, FRA; 5 Ophthalmology, Mediclinic City Hospital, Dubai, ARE; 6 Ophthalmology, Saint Joseph University, Beirut, LBN; 7 Ophthalmology, Beirut Eye & ENT Specialist Hospital, Beirut, LBN

**Keywords:** topography, prk, collagen cross-linking, post-lasik ectasia, keratoconus

## Abstract

Purpose

To report the response of keratoconus (KC) and post-LASIK ectasia (referred to as “ectasia”) to the corneal crosslinking (CXL) and to compare the rate of progression between KC and ectasia at three years.

Methods

A retrospective cohort study of patients undergoing CXL for either KC or ectasia. Fifty-four eyes (31 patients) with ectasia and 111 eyes (67 patients) with KC were included in the study. Corrected distance visual acuities (CDVA), refraction, keratometry (K), and pachymetry were followed up for three years. Simultaneous photorefractive keratectomy (PRK) and CXL were performed on 20 KC and 20 ectasia eyes. Intrastromal Corneal Ring Segments (ICRS) were performed on 51 KC and six ectasia eyes.

Results

In KC, CDVA, spherical equivalence, sphere, cylinder, and mean K improved at three years post-CXL (p-value<0.05), but these values improved without reaching a statistical significance in ectasia(p-values <0.05). 12 of 54 eyes with ectasia (22.2%) and 4 of 111 eyes (3.6%) with KC had progression post CXL(p-value:0.0001). Ectasia patients diagnosed with progression were older at presentation (36.1 years) than non-progressive ectasia patients (31 years) (p-value 0.02) and also older than KC patients.

Sub-analysis excluding PRK and ICRS cases showed that there was an improvement in mean sphere (from -5.23±4.2D to-4.46±3.89D) (p-value 0.03) cylinder (from 2.54 ± 1.68D to 1.97 ± 1.51D) (p-value 0.03) mean keratometry (from 46.81 ± 3.78D to 46.01 ± 3.25D) (p-value 0.006) in KC patients 3 years post CXL (40 patients). Compared to baseline, all the mean refractive and topographic variables deteriorated at three years post CXL in ectasia (28 patients) (p-value>0.05). Also, 2 of 40 patients with KC (5%) vs. 7 of 28 patients with ectasia (25%) had progression three years post-CXL, and the difference between both groups remained statistically significant(p-value 0.027).

Conclusion

Eyes with post-LASIK ectasia seem to be less responsive to CXL than KC.

## Introduction

Keratectasias are progressive, non-inflammatory corneal diseases defined by thinning, bulging, and distortion of the cornea leading to irregular astigmatism and reduction in vision [1]. Early progressive disease is treated with corneal crosslinking (CXL) to stabilize the cornea, while end-stage cases are treated with penetrating or deep anterior lamellar keratoplasty. Visual rehabilitation of stable corneas includes spectacle correction, hard contact lens or scleral contact lens, intrastromal corneal ring segment implantation (ICRS), phakic intraocular lens, and topography-guided photorefractive keratectomy (PRK) [1].

After introducing CXL with riboflavin in 2003, it became the primary treatment to reduce the keratoconus and post-LASIK ectasia progression [2]. Ultraviolet-A light induces a photochemical reaction in the corneal stroma in the presence of riboflavin, leading to a more covalent connection between collagen fibers and hence, stabilizing the cornea and improving collagen structure [3]. (For the sake of simplicity, we will be using from here on the term “ectasia” to signify post-LASIK ectasia).

Recently, PRK and ICRS insertion were combined with CXL (simultaneously or staged procedures) to improve the uncorrected and best-corrected visual acuity of the patients and strengthen their corneas simultaneously [4,5]. Multiple studies proved the effectiveness of CXL and reported good outcomes in terms of visual acuity, refraction, corneal curvature, and keratometry on the short and long-term follow-up in patients with keratoconus and ectasia [3,6-8]. However, many corneas failed to stabilize after CXL and had progressive steepening. The failure rate was variable between studies. In patients with keratoconus, it was reported to be between 3.17% and 33% [3,9-12]. On the other hand, in the smaller studies conducted on patients with ectasia, the failure rate ranged between 0 to 27.5% [13,14].

Ectasia and keratoconus responses to CXL were previously compared over one year. It was postulated that the reduced effect of CXL post-LASIK may be caused by the flap, which may inhibit the diffusion of riboflavin, or due to the anterior stromal behavioral change by the CXL process [15]. Most comparative studies were performed on a small sample of patients or for a short period [13-15]. Therefore, we decided to investigate the effect of CXL on keratoconus and ectasia groups simultaneously and to calculate the difference in the rate of progression between both groups on the long-term follow-up, in addition to the characteristics and the risk factors of CXL failure in each group.

This article was previously posted to the research square preprint (https://www.researchsquare.com/article/rs-239994/v1) server on March 11, 2021.

## Materials and methods

A retrospective cohort study was conducted at Beirut Eye and ENT Specialist Hospital (Lebanon) between January 2010 and June 2015. Patients who underwent CXL surgery for progressive keratoconus and ectasia were followed for three years. Institutional Review Board (IRB) approved the study, which complies with the Declaration of Helsinki (IRB approval number 2019-5).

Study population

Charts of all the patients receiving CXL between January 2010 and June 2015 were reviewed. One hundred fifty-eight patients with keratoconus and 62 patients with ectasia were identified.

Inclusion criteria: Progressive keratectasia (keratoconus or ectasia) based on refraction and topography changes in two consecutive visits (criteria discussed in the following section), patients who presented for follow up at six months, one year, and three years, the minimal central corneal thickness of 330 μm [16], all age groups.

Exclusion criteria: loss of follow-up, ocular surface pathologies, pregnancy during the three years of follow-up, eyes implanted with implantable Collamer lens (ICL) during the follow-up period. From the identified patients, 25 were excluded due to ICL implantation and 97 due to loss of follow up)

**Figure 1:**
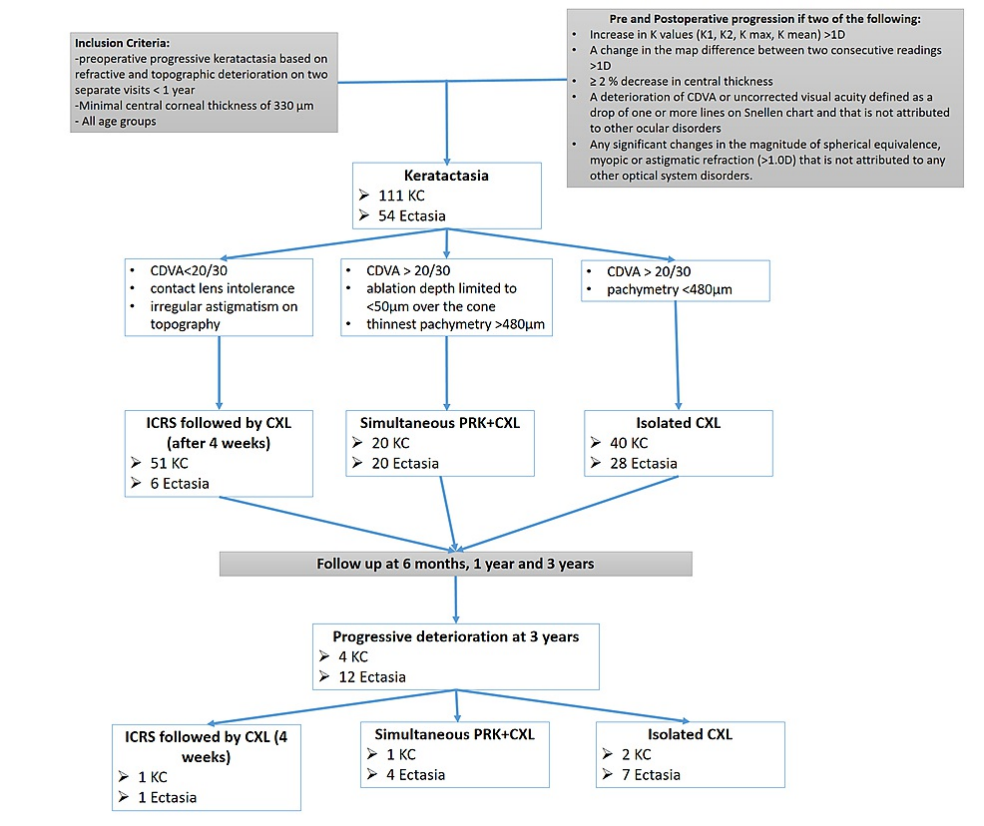
Inclusion criteria, pre, and postoperative patient’s distribution CDVA: Corrected Distance Visual Acuity; CXL: Corneal Crosslinking; D: Diopters; Ectasia: post-LASIK ectasia; ICRS: Intrastromal Corneal Ring Segments; K: Keratometry; KC: Keratoconus; PRK: Photorefractive Keratectomy

165 eyes of 98 patients met the inclusion criteria. Fifty-four eyes (31 patients) had ectasia, and 111 eyes (67 patients) had keratoconus. Since keratoconus is more prevalent than ectasia, a higher number of patients was included. In both keratoconus and ectasia groups, some patients were subject to combined procedures, ICRS or PRK. In order to avoid biased statistics, statistical analysis was first carried in all patients, comparing both groups. Then patients who had ICRS or/and PRK were then excluded from the final analysis comparing ectasia and CXL at different follow-up periods (Figure 1).

Keratoconus and ectasia progression

Progression in patients diagnosed with keratoconus and ectasia is suspected if there is deterioration in the visual acuity or the manifest refraction after increased maximum keratometry readings or decreased corneal thickness [12,17-19]. Until now, there is no clear definition of progression. The global consensus on Keratoconus and Ectatic Diseases (2015) defined progression by a change in at least two of the following parameters: progressive steepening of the anterior corneal surface, steepening of the posterior corneal surface or thinning or changes in the pachymetric rate of change [17].

In our study, the progression before and after CXL was diagnosed based on the presence of two or more of the following criteria on two consecutive visits: Increase in K values (K1, K2, K max, and K mean) >1D; A change in the map difference in K values between two consecutive readings >1D; ≥ 2 % decrease in central thickness; A deterioration of CDVA or uncorrected visual acuity is defined as a drop of one or more lines on the Snellen chart, and that is not attributed to other ocular disorders; Any significant changes in the magnitude of spherical equivalence, myopic or astigmatic refraction (>1.0D) are not attributed to any other optical system disorders.

Surgical procedure

CXL-epithelium off was used for all the surgeries. Proparacaine hydrochloride 0.5% drops were used to anesthetize the eye. After the insertion of a lid speculum, a blunt spatula was used to remove the central 9 mm corneal epithelium. The riboflavin 0.1% dextran solution (Collagex, isotonic 0.1%, Lightmed USA Inc.) was instilled every 2 minutes for 30 minutes. The ultraviolet A (UVA) lamp (UV-X illumination system, version 1000; IROC AG, Zurich, Switzerland) was then focused on the cornea with the radiant energy of 3.0 ± 0.3 mW/cm2 for 30 minutes following Dresden protocol [2]. During UVA administration, riboflavin drops were applied to the cornea every 2 minutes. The thinnest and central pachymetry were continuously monitored through the procedure using Accutome AccuPach VI 24-6200 Pachymeter Digital Signal Analysis (Accutome, Inc, USA). Eyes with a pre-operative central corneal thickness between 330 and 400 μm, application of hypo-osmolar riboflavin for 5 minutes (1 drop every 20 seconds) was performed until adequate corneal thickness was reached (>400 μm) [16].

After treatment, gatifloxacin 0.5% (Zymaxid®; Allergan, Inc.) eye drops were instilled, followed by the placement of a bandage soft contact lens (ACUVUE®, Johnson & Johnson Vision Care, Inc.) for five days. Postoperatively, patients were given gatifloxacin 0.5% four times daily for seven days, Tobramycin-dexamethasone 0.1% (TOBRADEX® Alcon Laboratories, Inc) four times daily for 10 days, followed by Loteprednol (LOTEMAX® Bausch & Lomb. Inc.) 0.5% five times daily, tapered over five weeks [10].

Some patients in both groups were subject to the combined procedures, ICRS, and PRK. ICRS (Intacs® Addition Technology™, Inc.) were implanted four weeks prior to CXL in both groups if the patient had a decreased CDVA (<20/30), contact lens intolerance, and irregular astigmatism on topography. A total of 57 eyes were implanted with ICRS using Intralase femtosecond laser (IntraLase, Abbott Medical Optics Inc., Santa Ana, California, USA) to create the tunnels at 400µm depth (different ring segment arc length and thickness were used following our published algorithm in each case) [20]. Also, a simultaneous PRK using an excimer laser (ALCON WAVELIGHT® EX500 Alcon Laboratories, Inc.) was performed at the same day prior to CXL if the patient had a CDVA > 20/30, a minimum pre-operative thinnest pachymetry of 480µm and a calculated ablation depth limited to <50µm over the cone. The 9 mm central corneal epithelium was removed using a blunt spatula. PRK was performed on 40 eyes (Figure 1).

Data collection and outcome measures

Patients’ charts were reviewed. Assessment of their uncorrected distance visual acuity, corrected distance visual acuity (CDVA), and manifest refractions using Snellen charts was performed preoperatively, six months, one year, and three years post-treatment. The logarithm of the minimum angle of resolution (logMAR) system was used to document and analyze visual acuity.

Also, slit lamp exam, fundus evaluation, and corneal topography maps were performed during each visit. The WaveLight® Allegro Oculyzer™ (WaveLight, GmbH, Erlangen, Germany) was used to record keratometry (K1, K2, K mean, K max) pachymetry.

Statistical analysis

SPSS 19.0 (SPSS, Inc, Chicago, IL) was used to analyze data. Continuous variables (visual acuity, spherical equivalence, keratometry, pachymetry) were analyzed as mean and SD (standard deviation), while categorical variables (gender, ICRS, PRK) were presented as percentages. The progression rate was calculated for both keratoconus and ectasia. Paired sample t-test was used to explore relationships between continuous variables, while the chi-square test (Fisher’s Exact Test) was used to compare categorical variables and progression rate. P-value was considered significant if less than 0.05.

## Results

Of the 165 eyes included in the study. One hundred eleven eyes of them (67 patients) had keratoconus with a mean age of 26.2 ±8.3 years (67 males and 44 females), and 54 eyes (31 patients) had ectasia with a mean age of 32.4 ± 7.8 years (26 males and 28 females).

It is well known that PRK and ICRS may be a source of bias due to the unpredictable changes of the tomographic parameters. Therefore, analysis of keratoconus and ectasia response to CXL at three years was performed with and without excluding simultaneous PRK or ICRS implantation cases.

Analysis including all patients

Keratoconus patients had progressive improvement of the mean CDVA over three years (improvement by 0.07 logMAR). Although the improvement is statistically significant, it is not clinically relevant. Also, spherical equivalence, sphere, cylinder, and mean keratometry decreased, reaching statistical significance after six months. The maximum keratometry was significantly different at three years. The thinnest pachymetry did not show any difference three years post CXL. Ectasia patients had minimal improvement in CDVA, spherical equivalence, sphere, cylinder, and mean keratometry, and results did not reach significance at three years post-CXL (p-value> 0.05). Only pachymetry deteriorated by 16.7µm at three years (p-value 0.04) (Table 1, 2).

**Table 1:** Pre and post-crosslinking refractive measurements (corrected distance visual acuity, spherical equivalence, sphere, and cylinder). CDVA: Corrected distance visual acuity; CI: confidence interval; CXL: collagen cross-linking.; KC: keratoconus; n: number of patients; ICRS: intrastromal corneal ring segments; PRK: photorefractive keratectomy p-value: using paired sample t-test comparing three years with pre-CXL data **significant changes with p-value <0.05 compared to pre-CXL data

Group	Factor	Patients	Pre-CXL	6 months Post-CXL	1 year Post-CXL	3 years Post-CXL	P-value (at 3years)	CI (at 3years)
Including all cases (with ICRS and PRK)	CDVA (logMAR)	KC (n=111)	0.16±0.21	0.15 0.14	0.1±0.11**	0.09±0.21**	0.001	0.04; 0.11
		Ectasia (n=54)	0.1±0.14	0.11 0.18	0.09±0.1	0.09±0.11	0.4	-0.02; 0.05
	Spherical equivalence (diopters)	KC (n=111)	-3.4±3.37	-2.39±3.57**	-2.39±3.63**	-2.38±3.8**	0.001	-1.48; -0.54
		Ectasia (n=54)	-2.64±3.73	-2.35±4.35	-2.49±4.09	-2.29±3.72	0.15	-0.82; 0.13
	Sphere (diopters)	KC (n=111)	-4.7±3.74	-3.37±3.92**	-3.36±3.98**	-3.29±4.14**	0.001	-1.92; -0.89
		Ectasia (n=54)	-3.5±3.75	-3.12±4.5	-3.24±4.25	-3.05±3.93	0.08	-0.96; 0.05
	Cylinder (diopters)	KC (n=111)	2.6±1.73	1.94±1.48**	1.93±1.59**	1.84±1.53**	0.001	0.45; 1.07
		Ectasia (n=54)	1.72±1.32	1.55±1.55	1.49±1.55	1.5±1.55	0.27	-0.17; 0.59
Excluding ICRS and PRK cases	CDVA (logMAR)	KC (n=40)	0.14±0.23	0.14±0.13	0.11±0.12	0.1±0.13	0.24	-0.02;0.09
		Ectasia (n=28)	0.11±0.12	0.13±0.23	0.12±0.12	0.12±0.12	0.35	-0.45;0.01
	Spherical equivalence (diopters)	KC (n=40)	-3.96±3.89	-3.36±3.77**	-3.22±3.66**	-3.48±3.71	0.11	-1.08;0.12
		Ectasia (n=28)	-4±4.58	-4.14±5.4	-4.58±4.68	-4.25±4.29	0.49	-0.48;0.98
	Sphere (diopters)	KC (n=40)	-5.23±4.2	-4.45±4.07**	-4.26±3.82**	-4.46±3.89**	0.03	-1.48;-0.04
		Ectasia (n=28)	-4.9±4.48	-5.09±5.4	-5.62±4.66	-5.26±4.31	0.3	-0.35;1.08
	Cylinder (diopters)	KC (n=40)	2.54±1.68	2.15±1.63	2.08±1.6	1.97±1.51**	0.03	0.05;1.08
		Ectasia (n=28)	1.79±1.53	1.9±1.65	2.07±1.68	2.02±1.76	0.41	-0.8;0.34

**Table 2:** Pre and post-crosslinking tomographic measurements (mean keratometry, maximum keratometry, and thinnest corneal pachymetry). CI: confidence interval; CXL: collagen cross-linking; K: keratometry; KC: keratoconus; n: number of patients; ICRS: intrastromal corneal ring segments; PRK: photorefractive keratectomy p-value: using paired sample t-test. **significant changes with p-value <0.05 compared to pre-CXL data

Group	Factor	Patients	Pre-CXL	6 months Post-CXL	1 year Post-CXL	3 years Post-CXL	P-value (at 3years)	CI (at 3years)
Including all cases (with ICRS and PRK)	K Mean (diopters)	KC (n=111)	47.46±4.4	46.4±4.23**	46.16±4.13**	46.1±4.12**	0.001	0.85; 1.7
		Ectasia (n=54)	44.07±3.29	43.38±3.75**	43.54±4.07	43.47±3.85	0.08	-0.08; 1.28
	K Maximum (diopters)	KC (n=111)	54.49±7.99	54.66±8.44	53.97±7.92	53.36±7.56**	0.001	0.49; 1.76
		Ectasia (n=54)	48.77±8.74	49.33±5.07	49.49±5.65	49.83±5.65	0.34	-3.28; 1.16
	Thinnest Pachymetry (µm)	KC (n=111)	462.5±45.38	455.8±7.99**	456.8±42.2**	458.5±41.37	0.13	-1.23; 9.39
		Ectasia (n=54)	464.3±60.72	446.7±72.85**	448.3±82.35	447.6±79.47**	0.04	0.14; 33.1
Excluding ICRS and PRK cases	K Mean (diopters)	KC (n=40)	46.81±3.78	46.23±3.36**	46.1±3.31**	46.01±3.25**	0.006	0.24;1.34
		Ectasia (n=28)	44.11±3.63	44.24±4.38	44.29±4.25	44.4±4.44	0.365	-0.94;0.35
	K Maximum (diopters)	KC (n=40)	52.14±6.63	52.51±6.98	52.1±6.79	51.7±6.42	0.26	-0.34;1.21
		Ectasia (n=28)	50.99±6.35	51.13±5.87	51.54±6.61	52.01±6.31	0.106	-2.26;0.23
	Thinnest Pachymetry (µm)	KC (n=40)	461.9±45.3	464.1±36.2	463.9±42.3	463.4±42.8	0.699	-8.97;6.08
		Ectasia (n=28)	463.1±57.9	448.8±74.3	459.5±69.4	460.8±59.9	0.553	-5.61;10.25

**Figure 2:**
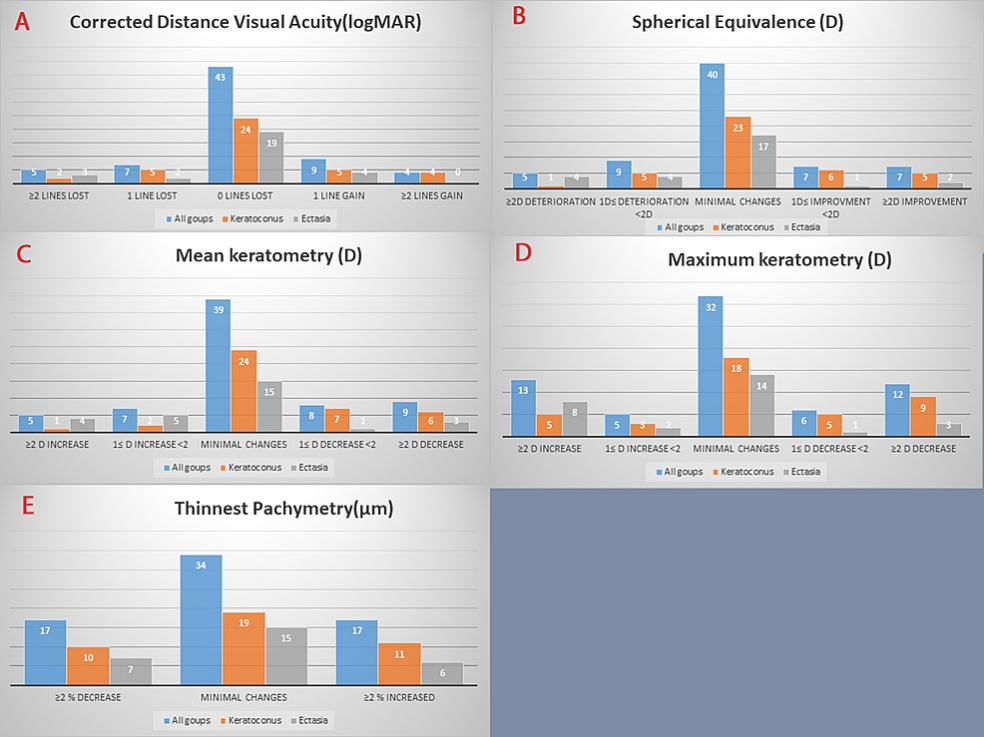
Changes in ‘’Corrected Distance Visual Acuity (A), spherical equivalence (B), mean keratometry (C), Maximum keratometry (D), thinnest corneal pachymetry (E)’’ between baseline and 3 years post-CXL. (Excluding PRK and ICRS cases). CXL: collagen cross-linking; K: keratometry; KC: keratoconus; ICRS: intrastromal corneal ring segments; PRK: photorefractive keratectomy

Analysis excluding PRK and ICRS patients:

At three years post CXL, the mean CDVA and spherical equivalent were not statistically different from baseline in keratoconus and ectasia groups, seven patients with keratoconus (17.5%) and five patients with ectasia (17.8%) lost one or more lines of CDVA. Also, six patients with keratoconus (15%) and eight patients with ectasia (28.5%) had a deterioration of 1 or more diopters of spherical equivalence (table 1 and figure 2).

Keratoconus patients had a progressive decrease in the mean sphere and cylinder up to 3 years post CXL (p-value 0.03). On the contrary, ectasia patients had a progressive increase in the mean sphere and cylinder without reaching significance (Table 1).

In keratoconus, the mean keratometry decreased significantly by 0.8 D at three years post-CXL (p-value 0.006). The maximum keratometry decreased by 0.44 D without reaching a statistically significant difference (p-value 0.26). In ectasia, mean and maximum keratometry increased, reaching a difference of 0.29D and 1.02 D, respectively, at three years compared to baseline (p-value>0.05) (Table 2).

Over three years of follow-up, the changes in the mean corneal thickness at the thinnest location were not statistically significant in keratoconus and ectasia. Ten patients with keratoconus (25%) and 7 with ectasia (25%) lost more than 2% of their corneal thickness during the follow-up period (table 2 and figure 2).

Keratoconus and post-LASIK ectasia progression post-CXL

Of the total 165 eyes included in the study, 111 eyes had keratoconus, of which four eyes (3.6%) had progression. Fifty-four eyes had ectasia, of which 12 eyes (22.2%) met the criteria for progression three years post-CXL. The difference was statistically significant (p-value 0.0001).

Patients diagnosed with progressive keratoconus at three years post-CXL were younger at the first presentation than patients with stable keratoconus (23±8.4 vs. 26.3±8.3 years respectively), but this was not significantly different (p-value 0.43; CI: [-5.1; 11.7]). Patients who had progression of their ectasia three years post CXL were older at presentation than those with a stable ectasia (36.1±6.4 vs. 31±7.7 years respectively), and this difference was significant (p-value 0.02; CI: [-10.7; -0.7]).

After excluding all the patients who received PRK or ICRS: 2 of 40 patients with keratoconus (5%) vs. 7 of 28 patients with ectasia (25%) had progression three years post-CXL, and the difference between both groups remained statistically significant (p-value 0.027). The nine patients with progression included in the subgroup analysis had two or more of the previously mentioned criteria indicating failure of CXL (2 patients with keratoconus and 7 with ectasia) (Table 3).

**Table 3:** Variables changes at 3 years of follow-up compared to baseline in each of the patients diagnosed with progression and failure of crosslinking (excluding cases with intrastomal rings and PRK). + indicates significant deterioration suspicious of progression according to our criteria Indicates improvement  or minimal changes compared to baseline CDVA: corrected distance visual acuity; K: keratometry; SE: spherical equivalence

Patient number	1	2	3	4	5	6	7	8	9
Pathology	ectasia	ectasia	ectasia	ectasia	ectasia	ectasia	ectasia	KC	KC
CDVA	-	+	-	-	+	+	-	-	+
SE	+	+	-	-	+	+	+	+	-
Sphere	+	+	-	+	+	+	+	+	-
Cylinder	-	-	+	+	+	-	-	+	-
Mean K	-	+	-	-	+	+	-	-	+
Max K	+	+	+	+	+	-	-	-	-
Thinnest pachymetry	+	+	-	+	-	-	-	+	-

Procedure complications

Of the total pool of patients, only one patient who operated with CXL without ICRS or PRK presented with late postoperative haze. Infectious keratitis or healing defect were not reported in our series. To note that 10 patients (8 with keratoconus and two with ectasia) had a central corneal thickness between 340 and 400, for which a hypo-osmolar riboflavin solution was used during the CXL procedure without any complication.

## Discussion

Until now, corneal cross-linking has been proved to be the most effective method used to halt the progression of keratoconus and other corneal ectatic diseases [12,21,22].

Multiple studies have shown long-term stability post CXL in keratoconus patients with minimal risk of progression [3,9-11]. Other newly published studies have shown relative stability post-CXL in patients with ectasia. In a US-based multicenter clinical trial of CXL for Treatment of post-Lasik ectasia, 91 patients who received CXL showed improvement of the maximum keratometry compared to the sham group at one year, 4% of the treated patients had significant deterioration of their maximum keratometry and CDVA [23]. In another report, 13 of the 17 treated eyes had stable or improved CDVA over an 80 month mean follow-up period [24]. In a study, 2 of 14 patients with ectasia had keratometric deterioration between one and three years of follow-up [13]. Moreover, another report showed that 27.5% of patients with ectasia had lost Snellen lines over two years [14].

On the other hand, few comparative studies have been published. Over a one-year follow-up, 3 of the 22 patients with ectasia lost two Snellen lines compared with 3 of 49 eyes with keratoconus [15]. The latter study found no difference between keratoconus and ectasia response to CXL [15]. Unfortunately, these studies were performed either on a small population or over a short period. Most of them were non-comparative and failed to identify the number of patients who met the criteria for progression after CXL, defined by the global consensus on Keratoconus and Ectatic Diseases. Therefore, comparing KC and post-Lasik ectasia is not accessible from different studies on different populations and different treatment protocols.

In our study, the progression rate post-CXL was significantly higher in ectasia patients compared to keratoconus over three years. To mention that progression was diagnosed at least one year after the CXL, because we consider that the response corneal remodeling after CXL is mostly achieved at six months post-surgery and the topography stabilizes at six months’ post-CXL, which is considered as a baseline data after CXL that will be used for further analysis. To explain the higher rate of ectasia progression after CXL, we hypothesize that progression in ectasia patients could be due to a small bio-mechanically effective residual stromal bed post-LASIK procedure that the anterior flap does not contribute to the biomechanical stability restoration after CXL [25]. On the contrary, the flap itself may form a barrier to hinder riboflavin and UV light penetration to the deeper residual stromal bed tissue [15]. Hence, photosensitization of the deep stromal tissue in the residual stromal bedpost LASIK happens at a much lower effective rate than in the upper part of the stromal tissue in keratoconus during the CXL procedure.

Moreover, the histopathological and ultrastructural differences between ectasia and keratoconic corneas have been previously described [26]. In a study, both pathologies were similar in terms of having fewer and thinner than regular lamellae in the region of ectasia. However, only the residual stromal bed in post- LASIK corneas showed these changes while all the corneal thickness was affected in keratoconus [26]. Moreover, since the anterior corneal stroma -which represents the strongest region- has been ablated by the flap, the cornea post- LASIK was expected to respond differently to the biomechanical treatments as those used in ICRS placement or CXL [26].

Our analysis showed that post-LASIK ectasia patients were older. This could explain the progressive deterioration of these corneas over a long period, reaching a threshold of clinical significance at older ages. However, the date of their refractive surgery is unknown to prove this theory.

PRK procedure, along with CXL, has been proved to be safe and effective in patients with keratoconus [27]. In this study, the rate of performed PRK in progressive and non-progressive cases (in keratoconus and ectasia groups) were comparable. But, because of a possible selection bias, no conclusion can be drawn concerning the safety of PRK in keratectasia and its contribution to the progressive deterioration of some cases.

In the subgroup analysis excluding patients who received PRK or ICRS: the mean CDVA, SE, mean and maximum keratometry had continuous improvement from six months’ post-CXL up to three years in keratoconus patients (though not always reaching statistical significance or clinical significance). On the other hand, the mean CDVA, SE, mean and maximum keratometry deteriorated in patients with ectasia at three years post-CXL. These results are in concordance with the published literature about the long-term outcomes of CXL in keratoconus patients [28].

The significance of the changes in CDVA and refractive outcomes at three years compared to baseline decreased after eliminating PRK and ICRS cases in keratoconus patients. This suggests that the changes reported in the total population were not caused by the crosslinking. They are most likely the result of the ICRS, which is known to improve the CDVA and decrease the spherical equivalence.

In our study, we tried to correlate the amount of historical LASIK ablation depth and the original refraction to the progression of ectasia. However, the data was nonexistent in most of the patients since it was performed in other centers, and it could not be concluded from the available data due to ectasia. The changes in maximum keratometry were more indicative of progression in ectasia patients than keratoconus patients. A possible explanation for this difference can be attributed to keratoconus being a disease that affects the entire cornea, while ectasia is more localized. Moreover, a proposed treatment using deep stromal puncturing may potentially restore the flap’s contribution to the biomechanical corneal stability in post-Lasik ectasia patients [29].

There are some limitations to the current study. First, it was performed in a single-center, and the surgeries were conducted by the same surgeon. Second, the number of patients decreased after excluding ICRS and PRK patients. Third, a selection bias is present since many patients with keratoconus and post-Lasik ectasia were lost to follow-up and were excluded from the study.

## Conclusions

In conclusion, CXL was proved effective in halting the progression of both post-Lasik ectasia and keratoconus. This article adds to the previous reports that post-LASIK ectasia patients had lower stability following CXL than keratoconus. Even though post-LASIK ectasia patients had non-significant changes in the mean refraction and topographic parameters post CXL, older patients and males with post-LASIK ectasia showed higher disease progression rates than younger patients. The positive effect of the CXL on the cornea can continue up to three years following Crosslinking surgery. However, many ectasia patients showed worsening of the refractive parameters as soon as six months following crosslinking.
